# Use of modelling tools to assess climate change impacts on smallholder oil seed yields in South Africa

**DOI:** 10.1371/journal.pone.0301254

**Published:** 2024-05-07

**Authors:** Priscilla Ntuchu Kephe, Siyabusa Mkuhlani, Farirai Rusere, Abel Chemura

**Affiliations:** 1 Potsdam Institute for Climate Impact Research (PIK), Member of the Leibniz Association, Potsdam, Germany; 2 Risk and Vulnerability Science Centre, University of Limpopo, Polokwane, South Africa; 3 International Institute of Tropical Agriculture, c/o ICIPE, Nairobi, Kenya; 4 School of Animal, Plant and Environmental Sciences, Faculty of Science, Wits Rural Knowledge Hub, Research Office, University of Witwatersrand, Johannesburg, South Africa; 5 Natural Resources Department, Faculty of Geoinformation Science and Earth Observation, University of Twente, Enschede, Netherlands; Government College University Faisalabad, PAKISTAN

## Abstract

Oil seed crops are the second most important field crops after cereals in the agricultural economy globally. The use and demand for oilseed crops such as groundnut, soybean and sunflower have grown significantly, but climate change is expected to alter the agroecological conditions required for oilseed crop production. This study aims to present an approach that utilizes decision-making tools to assess the potential climate change impacts on groundnut, soybean and sunflower yields and the greenhouse gas emissions from the management of the crops. The Decision Support Tool for Agrotechnology Transfer (DSSAT v4.7), a dynamic crop model and the Cool Farm Tool, a GHG calculator, was used to simulate yields and estimate GHG emissions from these crops, respectively. Four representative concentration pathways (RCPs 2.6, 4.5, 6.0, and 8.5), three nitrogen (0, 75, and 150 kg/ha) and phosphorous (0, 30 and 60 P kg/ha) fertilizer rates at three sites in Limpopo, South Africa (Ofcolaco, Syferkuil and Punda Maria) were used in field trials for calibrating the models. The highest yield was achieved by sunflower across all crops, years and sites. Soybean yield is projected to decrease across all sites and scenarios by 2030 and 2050, except at Ofcolaco, where yield increases of at least 15.6% is projected under the RCP 4.5 scenario. Positive climate change impacts are predicted for groundnut at Ofcolaco and Syferkuil by 2030 and 2050, while negative impacts with losses of up to 50% are projected under RCP8.5 by 2050 at Punda Maria. Sunflower yield is projected to decrease across all sites and scenarios by 2030 and 2050. A comparison of the climate change impacts across sites shows that groundnut yield is projected to increase under climate change while notable yield losses are projected for sunflower and soybean. GHG emissions from the management of each crop showed that sunflower and groundnut production had the highest and lowest emissions across all sites respectively. With positive climate change impacts, a reduction of GHG emissions per ton per hectare was projected for groundnuts at Ofcolaco and Syferkuil and for sunflower in Ofcolaco in the future. However, the carbon footprint from groundnut is expected to increase by 40 to 107% in Punda Maria for the period up to 2030 and between 70–250% for 2050, with sunflower following a similar trend. We conclude that climate change will potentially reduce yield for oilseed crops while management will increase emissions. Therefore, in designing adaptation measures, there is a need to consider emission effects to gain a holistic understanding of how both climate change impacts on crops and mitigation efforts could be targeted.

## 1. Introduction

Oilseed crops are a cornerstone and a significant source of human [1] and animal nutrition [2]. They are ranked the second most important field crops in total area planted, production, and consumption after cereals [3]. They are also used in producing multiple industrial materials and products [4] and biodiesel production [5]. Oil seed crops also improve soil fertility through nitrogen fixation [6, 7]. The increasing global population growth, urbanization, and transition to diets higher in refined oils or fats have also increased demand for edible oils and other by-products; hence oil seed crops have become pivotal for ensuring sustainable food security across the globe.

Many oil seed crops are grown across Africa; however, these crops are often underutilized, orphaned, or neglected partly as a result of a lack of knowledge about their nutritional and economic value. For example, Kephe et al. [8] and Caldas et al. [9] are of the opinion that the uncertainty of financial returns tends to steer farmers away from certain crops, especially if the crop is considered new and therefore unknown to them. Production of these oil seed crops is being threatened by climate change, raising fears of severe food insecurity [10]. In addition, the production of these crops has persevered with little formal support in resource-constrained farming systems such as those found in smallholder farming systems in South Africa [8] and SSA at large. This may suggest two things. First, they can potentially be resilient and possess certain desirable traits which may be useful for climate change adaptation. Secondly, they are on the brink of total collapse due to climate change, as little attention is paid to their resilience. Among the most important oil seed crops in Africa are soybean [*Glycine*. *max* (L.) Merr.], groundnut [*Arachis hypogaea* L] and sunflower [*Helianthus annus* L].

Soybean is an important crop for at least one million smallholder farmers in Africa and has considerable potential to mitigate soil fertility decline, enhance household food and nutrition security, increase rural incomes and thus reduce poverty [11]. Groundnut (peanut) is another important multi-purpose oil seed crop widely cultivated in sub-Saharan Africa (SSA) for its edible oil and confectionery uses. Sunflower is also an important oil seed crop that is considered to have high potential oil output and drought tolerance. The oil content of most of these oil seed crops ranges from at least 20% for soybeans to over 40% for sunflower [12]. Despite the critical importance of oil seed crops, yields in SSA are perennially low and may be further lowered by increased climate variability and change [8].

Most oil seed crops, such as sunflower, need a relatively shorter growing season, translating to low crop water requirements [13, 14]. They are, therefore, suitable for low rainfall conditions. The International Panel for Climate Change (IPCC) has predicted that at the current rate of emissions, atmospheric CO_2_ concentration might increase up to 660 ppm by 2060 and to 790 ppm by 2090 from the current 400 ppm [15]. In SSA, the mean average temperature has increased by 1.5°C from 1960 to 2000 and projections show an increase of 1.2°C to 3.4°C by 2060. Precipitation has been reducing since 1960 and this has been characterized by high variability through frequent droughts, floods and mid-season dry spells and the pattern is projected to continue [16]. Most of the farming in SSA is under rainfed conditions, with irrigated areas accounting for a very small proportion of agricultural output. The cost of irrigation is projected to rise due to the increased need to irrigate due to reduced rainfall reliability and high temperatures [17].

Considerable research on the impact of climate change on oil seed crops has been undertaken in Asia and Europe but not in Africa. In Asia, an average increase of 1°C and 2°C would reduce oil seed crop yields by 15 and 25% by late 2025 and 2050 respectively [18]. The limited climate change research on oil seed crops in SSA is focused much on West Africa [19]). The limited research that has been done is on crop breeding and agronomy in SSA [20] Across most of SSA, oil seed crops have been neglected in research, extension work, smallholder farming and policy planning. Poor yields and quality, price volatility, and unstable regulated markets have further reduced the attractiveness of oil seed crops among smallholder farmers [8, 21]. The study, therefore, sought to add to the limited body of knowledge on oil seeds (soybean, groundnut, sunflower) research specifically on climate change impacts and mitigation. This was undertaken by evaluating the potential impact of projected climate change on crop yields and carbon footprint of oil seed crops such as soybean, sunflower and groundnut in semi-arid regions of South Africa using modeling-based approaches. This study provides an entry point for further research related to climate change, yields, and GHG emissions of oil seed crops in Southern Africa and SSA at large.

## 2. Materials and methods

### 2.1 Study sites

**Fig 1:**
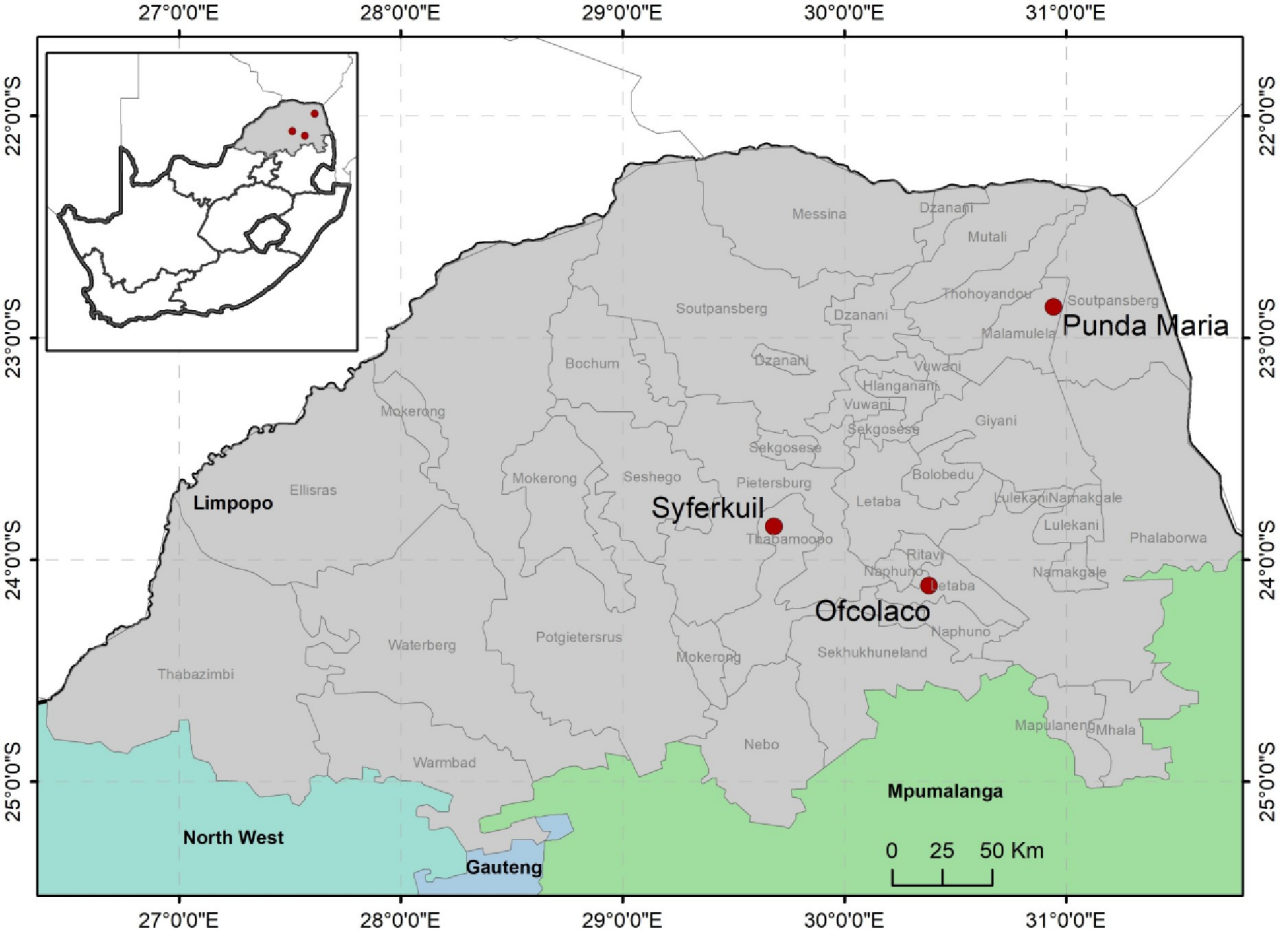
Punda Maria, Syferkuil and Ofcolaco, Limpopo province, the study areas, location of the experimental sites and weather stations.

The study was based on field experiments that were conducted during the summer growing seasons of 2016/2017 and 2017/2018, across three sites (Punda Maria, Ofcolaco and Syferkuil) in the Limpopo province, South Africa (Fig 1). The province has three distinct climatic regions that can be classified as (i) lowveld (arid and semi-arid) regions, (ii) middle veld and highveld (semi-arid) region, and (iii) the escarpment region which has a sub-humid climate receiving 700 mm rainfall per annum [22]. The climatic variation experienced in Limpopo allows this province to produce a variety of agricultural products such as tropical fruits, cereals, grains, legumes, and vegetables. Agricultural production in the province is diverse, but most smallholder farmers focus on crop production. Crop production highly depends on the summer rainfalls received mostly from October to March. Most smallholder farming is rainfed. In the first season, experiments were established at the Syferkuil experimental farm (23°50’38” S and 29°41’13” E) and at a farmer’s field at Ofcolaco (24°06’41” S and 30°23’26” E). In addition to these field trials, a farmer’s field at Punda Maria (22°49’18” S and 30° 54 ‘37” E) was included in the second season. Permission for data collection was facilitated following the granting of an ethical clearance from the University of Limpopo ethics committee (Faculty approval of proposal NO.89/2017) and by the signing of a consent form by the farmers. Daily rainfall, temperature, and solar radiation were collected from close meteorological stations during the 2016/2017 and 2017/2018 seasons.

### 2.2 Field experiments

#### 2.2.1 Soil data collection

Prior to the establishment of the field experiments, simple random sampling was used to collect soil samples with an auger to a depth of 0–90 cm at each location. The specific sampling depths were 0–15 cm and 15–30 cm, 30–60 cm and 60–90 cm. A total of 10 soil samples per experimental site were collected with a distance of 10m between sample points. The soil samples across all the locations were analyzed in the laboratory for various physical and chemical properties (Table 1).

**Table 1:** Soil physical and chemical properties of the experimental sites at Syferkuil, Ofcolaco and Punda Maria, Limpopo, South Africa that were used for the experiments.

Properties/Site	Syferkuil				Ofcolaco				Punda Maria			
Level (cm)	0–15	15–30	30–60	60–90	0–15	15–30	30–60	60–90	0–15	15–30	30–60	60–90
pH	6.7	6.4	6.0	6.3	5.9	5.2	5.4	4.9	6.42	6.3	7.1	5.5
Phosphorous (mg kg^-1)^	26.9	24.2	18	10	24.2	7.1	8.5	4.1	15.7	9.17	3.55	1.17
Potassium (mg kg^-1^)	464.3	325	163	159	231.6	165.9	142.8	78.6	264.3	325	163	159
Calcium (mg kg^-1^)	1215.8	1200	1222	1245	752.1	652.8	723.8	562.5	2700	1270	1212	879
Magnesium (mg kg^-1^)	699.8	709.8	809.8	999.8	298.8	291.2	346.4	154.4	712.4	809.8	539	599.8
Zinc (Zn)mg kg^-1^	2.78	2.2	1.6	0.8	3.7	3.1	1.5	0.4	2.8	2.2	1.6	1.1
Manganese (mg kg^-1^)	34.3	31.4	18	15	10.1	11.2	8.1	4.9	30.4	27.3	19.9	17.7
Copper (mg kg^-1^)	5.7	5.1	4.3	3.8	4.2	3.6	3.8	3.9	5.7	5.1	4.3	3.8
Total nitrogen (%)	0.19	0.06	0.04	0.1	0.04	0.04	0.04	0.04	0.09	0.05	0.04	0.3
Organic carbon (%)	0.89	0.8	0.6	0.5	1	0.6	0.8	0.8	0.91	0.72	0.3	0.02
Clay (%)	30	31	31	34	24	29	29	31	20	14	10	15
Silt (%)	7	8	12	10	9	10	11	11	20	31	30	30
Sand (%)	63	61	57	56	67	61	60	58	60	55	60	55
Textural class	Sandy clay loam				Sandy clay loam				Loam			

#### 2.2.2 Experimental design

The field experiments were laid out in a randomized complete block design (RCBD) with three replications and with experimental units measuring 3m x 3m. The treatments were different fertilizer application rates consisting of 0, 30, 60 kg/ha of phosphorus for soybean and groundnut applied as superphosphate at planting. For sunflower, the fertilizer treatment consisted of 0, 75, and 150 N kg/ha, applied as ammonium sulphate ((NH₄) ₂ SO₄) in split dose, at planting and at knee height. Experimental plots were kept weed free through manual weed control throughout the growing period using a hand hoe. Selected sunflower, soybean and groundnut cultivars were planted manually following the local production guidelines [23]. Soybean was planted at 75*10cm intra and inter-row spacing resulting in 133 333 plants ha^-1^. Sunflower was planted at 90*30cm inter and intra-row spacing resulting in 37 037 plants ha^-1^ and groundnut, at 60*30cm inter and intra row resulting in 55 555 plants ha^-1^. Planting and fertilizer applications were done on receipt of at least 20 mm and prediction of further rainfall within the next seven days afterward. Grain yield was harvested at maturity. Grain yield was determined by threshing all the pods from the harvested samples and drying the seeds to moisture content to constant weight. A digital balance scale was used to weigh the dried samples.

### 2.3 Crop model

The Decision Support System for Agro-technology transfer (DSSAT 4.7) model was calibrated based on the biophysical data from the on-station and on-farm research sites. The model is a dynamic and deterministic crop simulation model that has been used by researchers, governments and private organizations worldwide in over 100 countries [24] It is therefore a widely used process-based biophysical crop model that simulates crop growth as a function of soil-plant-atmosphere dynamics. The model requires soil surface and profile information, detailed crop management information, daily weather data, and plant varieties as inputs to produce plant and soil water, nitrogen, phosphorus, and carbon balances, as well as the vegetative and reproductive development at a daily time step [25]. The DSSAT v4.7 shell houses 28 independent crop models, each of which is calibrated before use. The DSSAT-CROPGRO model is one of the individual crop models within the DSSAT shell (www.dssat.net). CROPGRO model simulates the growth, development and yields of legume crops such as soybean, groundnut and sunflower. The model was calibrated based on the biophysical data collected from on-station and on-farm trials in Syferkuil, Ofcolaco and Punda Maria, Limpopo, South Africa. In this study, the DSSAT crop model was calibrated based on grain yield and days to flowering across the 3 crops and 3 sites for 2016/17-2017/18 seasons. The model performance was evaluated using the RMSE [26], which compares the measured and observed values. Specifically, RMSE values between 20–30% is considered ‘fair’, 10–20% ‘good’ and 0–10% is ‘excellent’ [26]. The model used actual CO_2_ values, based on the NOAA Mauna Loa, Hawaii measurements [27]. The corresponding CO_2_ values are also incorporated in the CMIP5 climate change scenarios used in this study. They were incorporated as the changes in temperature, rainfall and solar radiation computed based on the corresponding projected changes in greenhouse gasses which include CO_2_ and methane [28].

### 2.4 Climate change impact assessment

The study used projected climate data from CMIP5 [28] to assess the impact of climate change on the three crops across the three sites. The statistically downscaled data was sourced from the CGIAR-CCAFS program (http://ccafs-climate.org/data_bias_correction/http://ccafs-climate.org/data_bias_correction/) and bias corrected using the delta method [29]. The study used CMIP5, as this was the more publicly available future climate change data. The study used the representative concentration pathway (RCP) climate scenarios. RCPs are greenhouse gas concentration trajectories adopted by the IPCC as a future potential plausible state of the atmosphere. The RCPs are RCP 2.6, RCP 4.5, RCP 6, and RCP 8.5, which indicate the possible range of radiative forcing by the year 2100 with 2.6, 4.5, 6, and 8.5 W/m^2^, respectively. RCP 2.6 denotes a scenario of high efforts to reduce greenhouse gas (GHG) emissions and the RCP 4.5 denotes sub-medium efforts to curb GHG emissions leading to small increases in extreme weather conditions. RCP 6.0 denotes medium efforts to lower GHG emissions. RCP 8.5 denotes very low efforts to reduce GHG emissions which leads to large increases in extreme weather conditions. Such GHGs include methane, carbon dioxide, nitrous oxides and others. For this study data on climate change projections were accessed from the CCAFS climate data portal (http://www.ccafs-climate.org). The study simulated the yield of the three crops at three sites and fertilizer levels for the period 2015–2050. This window was selected to align the results with the Paris Agreement targets. The period until 2050 was used for 2 reasons, first to align results with Nationally Determined Contributions of Paris Agreement that set 2050 as target year. Second, to allow for results to influence medium term (10–25 years) planning as implementation of adaptation measures take time [30].

### 2.5 The cool farm tool

The Cool Farm Tool (CFT) is a model that estimates GHG emissions in both crop and livestock systems. It incorporates modules that consolidate many of the globally determined empirical models of GHG emissions into a GHG calculator [31]. The modules consist of a generic set of empirical models that are used to estimate GHG emissions based on a mix of IPCC Tier 1, Tier 2, and simple Tier 3 approaches. The model recognizes context-specific factors that influence GHG emissions such as pedo-climatic characteristics, production inputs, and other management practices at the farm level. The tool also has a strong farm-scale focus and was recently applied by Rusere et al. [32] in the estimation of GHG emissions from small-scale farming systems in South Africa. The CFT allows evaluation of the performance of cropping systems at the farm level in terms of both land and land use efficiency. Its detailed crop sub-module, which can account for land-use changes, fertilizer applications, and management changes such as tillage or cover cropping, fits the study’s ambition of evaluating the GHG emissions in cropping systems of the three oil seed crops.

#### 2.5.1 Assessing GHG emissions with the CFT at the field level

The CFT requires the following information to estimate GHG emissions from cropping systems (i) location, climate, soil parameters (soil moisture, drainage, pH, soil organic matter); (ii) material and energy inputs to farming, e.g., fertilizer and pesticide types and amounts and energy used on-farm and (iii) crop yields and harvested area. Data for characteristics of the study area were obtained from Kephe [33]. Soil characteristics data were measured from the study sites (Table 1). Fertilizer rates for the three treatments described above were used as input parameters into the models (Section 2.2.2). Tractor-drawn implements e.g., plough and discs were considered for land preparation. Oil seed crop yields for each of the sites were input into the CFT to compute the emissions per unit of land area.

#### 2.5.2 Calculation of greenhouse gas emissions at the field level

The above-mentioned data and crop yield from DSSAT were coupled into the CFT to calculate the GHG emissions of the three crops at the farm level. In the estimation of GHG emissions, a boundary was set to estimate emissions from the field only. Within the set boundaries, crops, soil inputs applied, fuel, and energy were used to estimate the GHG emissions for each crop per hectare. The irrigation and transport module components of the CFT were not included in the calculation of GHG emissions. This is because in these study areas crop production in small-scale farming systems is under rainfed conditions and transport was not included as we were only interested in GHG emissions at the field level. The study accounted for GHG emissions related to crop management and did not account for processing or transport beyond the farm gate. The carbon footprint of the three crops under study was then calculated by dividing the simulated GHG emissions per hectare by the simulated yields. The carbon footprint per unit yield (in kg CO_2_eq/kg) for each crop was calculated as:

$$CF_{per\;unit\;yield}=\frac{CFper\;unit\;area}{Yield}$$ (1)

The study evaluated the impacts of the four RCPs on GHG emissions of soybean, groundnut and sunflower per unit crop yield across different fertilizer regimes of 0, 30, and 60 P kg/ha for soybean and groundnut and 0, 75 and 150 N kg/ha for sunflower for the period 2015–2050.

## 3. Results

An analysis of the temperature data shows that mean minimum temperature increases from RCP 2.6 to RCP 8.5 for all the locations with Punda Maria having the highest increases. Punda Maria and Ofcolaco, also show gradual increases in maximum temperature as opposed to Syferkuil which shows a gradual decrease in temperature. Precipitation is generally lower at Punda Maria compared to other locations, with precipitation being low in the RCP 2.6 and 8.5, and relatively RCP 4.5 and 6.0 (Table 2). Rainfall tends to decrease across all locations and scenarios, with rainfall reduction being higher under RCP 8.5. Punda Maria has relatively low rainfall reduction rates compared to other areas. The reduction rates however are similar across the different emissions at 0.3 to 0.47 (Table 2).

**Table 2:** Changes in rainfall, minimum and maximum temperatures for Ofcolaco, Syferkuil and Punda Maria in South Africa for the period 2015–2050.

Emission scenario	Location	Mean Tmin	Mean Tmax	Average TotPrec	Change in Prec	Change in Tmin	Change in Tmax
RCP 2.6	Syferkuil	14.11	20.00	633.51	-0.32	0.013	0.0045
RCP 2.6	Ofcolaco	15.95	19.44	757.63	-0.34	0.013	0.0044
RCP 2.6	Punda Maria	18.49	19.21	285.04	-0.30	0.012	0.0043
RC P4.5	Syferkuil	15.38	20.10	642.60	-0.96	0.048	0.0118
RCP 4.5	Ofcolaco	17.20	19.54	769.93	-1.20	0.047	0.0115
RC P4.5	Punda Maria	19.66	19.31	291.11	-0.38	0.044	0.0114
RCP 6.0	Syferkuil	15.43	20.04	656.10	-0.80	0.065	0.0073
RCP 6.0	Ofcolaco	17.25	19.48	785.24	-0.85	0.064	0.0072
RCP 6.0	Punda Maria	19.67	19.25	302.77	-0.47	0.062	0.0070
RCP 8.5	Syferkuil	16.96	19.48	620.57	-1.31	0.127	0.0072
RCP 8.5	Ofcolaco	18.77	19.48	742.37	-1.48	0.126	0.0072
RCP 8.5	Punda Maria	21.91	20.73	283.13	-0.42	0.117	-0.0002

### 3.1 Experimental field trial yields

**Fig 2:**
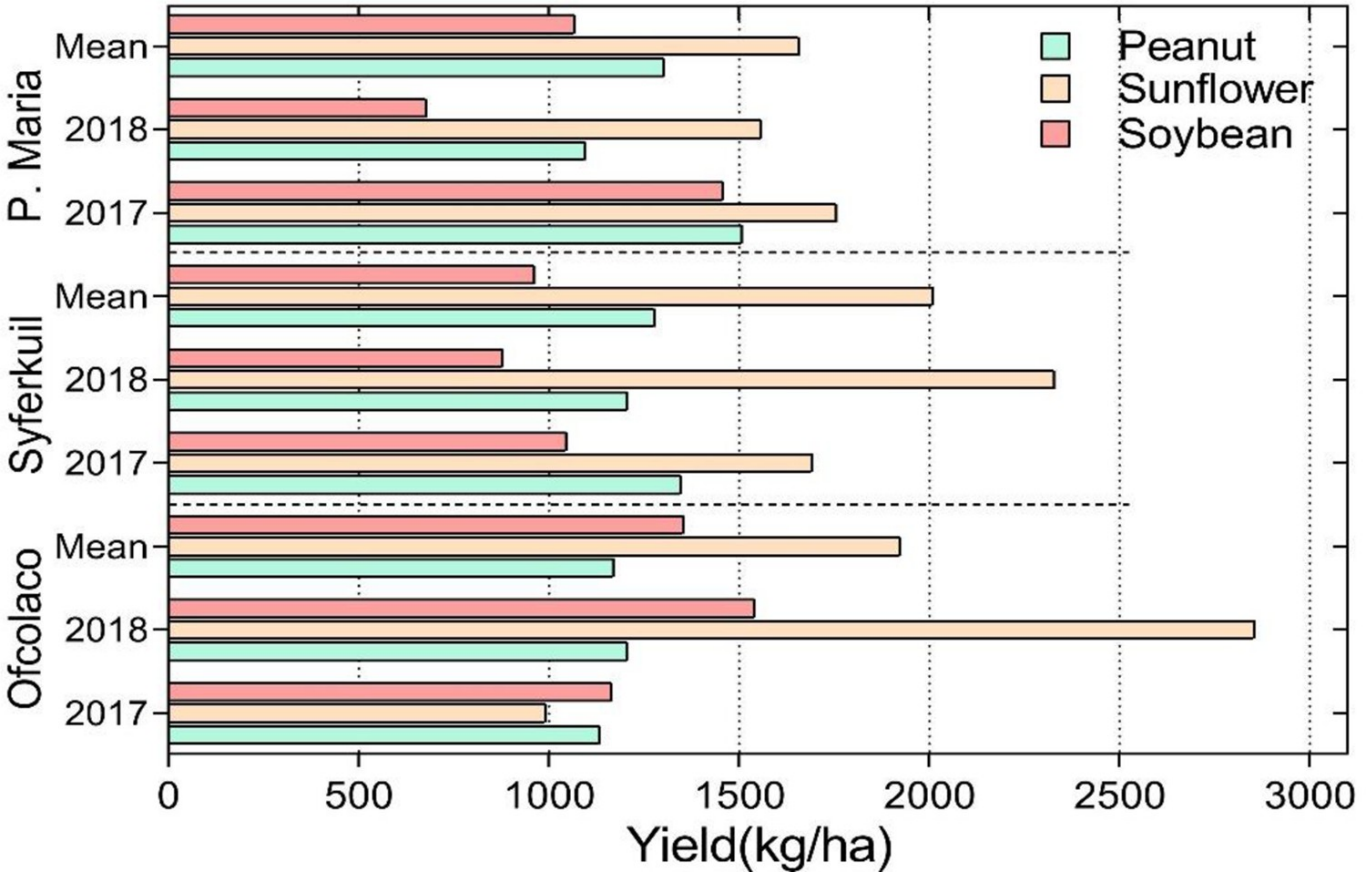
Groundnut, sunflower and soybean yields across the three experimental sites in Limpopo, South Africa during the period, 2016–2018.

Sunflower attained the highest yields across all sites and seasons, indicating it was the most productive of the three oil crops. The overall average yield for sunflower was 1864 kg/ha, which was 50% and 65% more than the yield of groundnut and soybean respectively under similar conditions. At Ofcolaco and Punda Maria, the highest yield was obtained in 2017 compared to 2018 while the opposite was the case at Ofcolaco, where yield was highest in 2017 compared to 2018 for all crops (Fig 2).

### 3.2 Model calibration and evaluation

In this study, the RMSE for at least 70% of the yield values across the 3 crops and 3 sites were less than 30% which is in the acceptable range (Table 3). For Ofcolaco, the RMSE was less than 30% for all crops and fertilizer levels but groundnut had an RMSE value between 20–30%. There were 4 scenarios in Syferkuil where the RMSE value was above 30%. At least 50% of the RMSE values were ’unacceptable’ in Punda Maria.

**Table 3:** Root mean square error (RMSE) values comparing measured and model simulated grain yields across the different crops and sites for the seasons, 2016/17-2017/18.

Crop	Fertilizer scenario (kg/ha)	Syferkuil RMSE (%)	Ofcolaco RMSE (%)	Punda Maria RMSE (%)
Sunflower	0 N	53.1	27.0	12.1
	75 N	60.4	5.4	2.5
	150 N	51.6	8.7	17.9
Soybean	0 P	16.6	7.0	64.7
	30 P	14.3	11.7	72.2
	60 P	27.0	11.7	68.1
Groundnut	0 P	29.4	29.7	27.1
	30 P	36.0	29.7	33.7
	60 P	27.7	28.5	33.9

### 3.3 Modeled climate change impacts

#### 3.3.1 Groundnut

**Fig 3:**
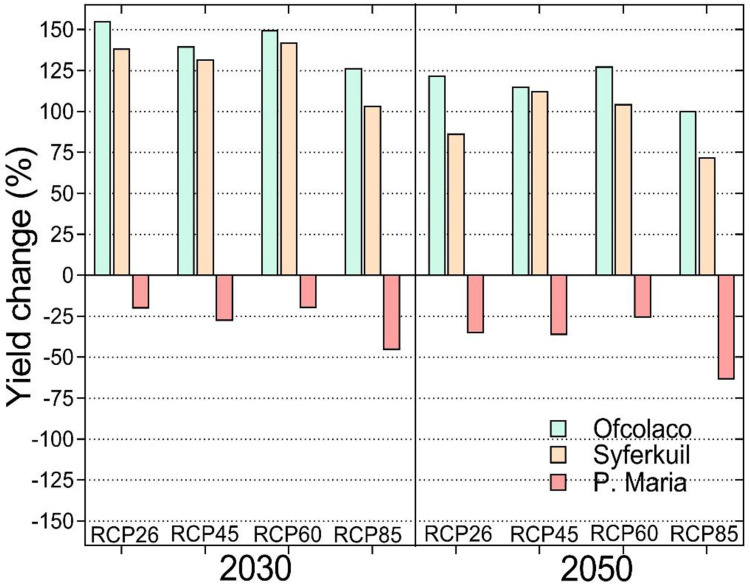
Predicted climate change impacts on groundnut yield at Ofcolaco, Syferkuil and Punda Maria, Limpopo, South Africa by 2030 and 2050 under four climate change scenarios.

Positive climate change impacts are predicted for groundnut at Ofcolaco and Syferkuil by 2030 and 2050 while negative impacts are projected at Punda Maria for the same period. The results project a doubling of yield under all four RCP scenarios by 2030 and lower but positive responses by 2050 in both Ofcolaco and Syferkuil (Fig 3). Compared to the positive impacts at other sites, yield losses of up to 50% are projected under RCP8.5 by 2050 in Punda Maria.

#### 3.3.2 Soybean

**Fig 4:**
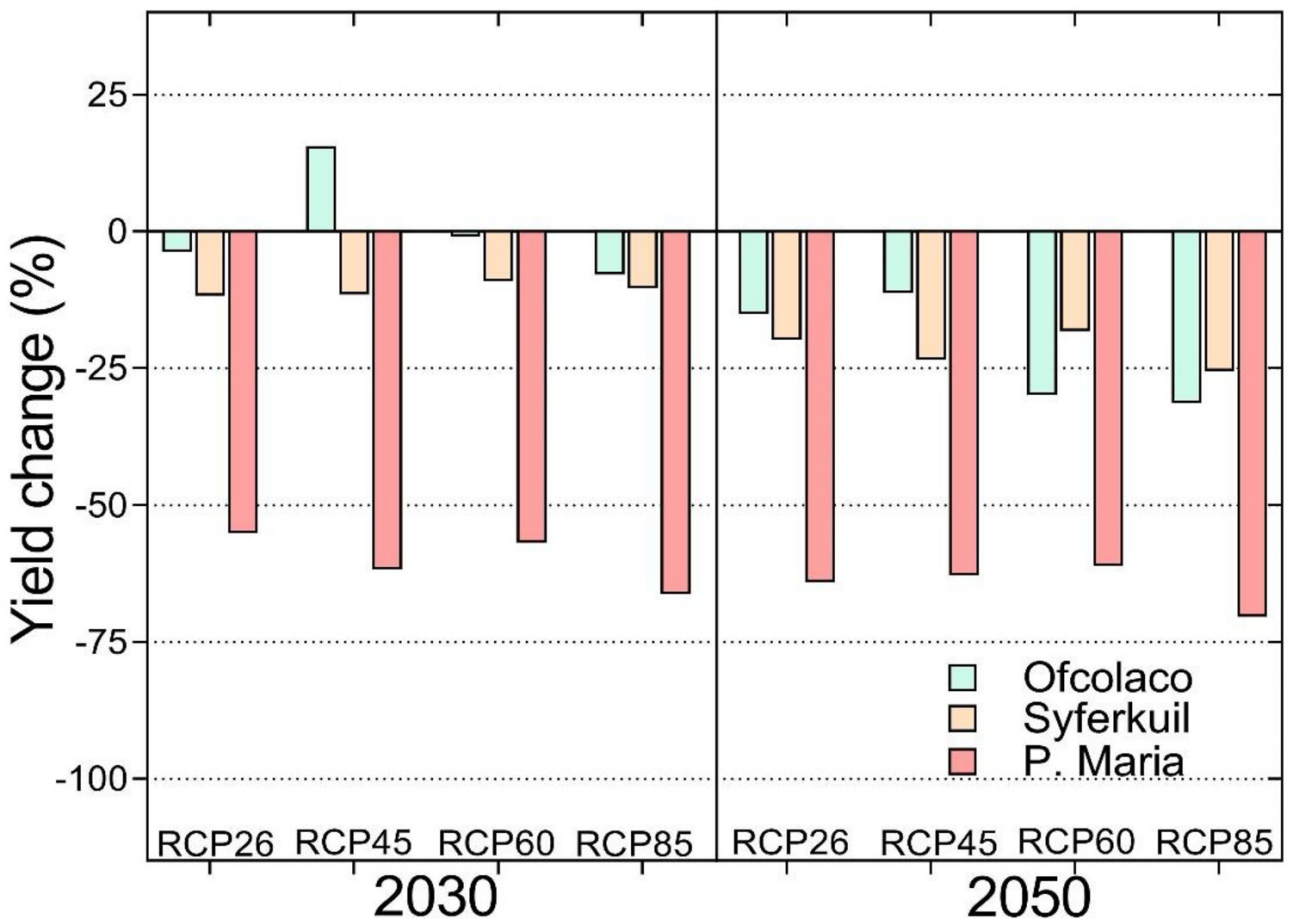
Predicted climate change impacts on soybean yield at Ofcolaco, Syferkuil and Punda Maria, Limpopo, South Africa by 2030 and 2050 under four climate change scenarios.

The yield of soybean is projected to decrease across all sites and scenarios by 2030 and 2050, except at Ofcolaco where under RCP 4.5 yield increase of 15.6% is projected. The worst climate change impacts are projected at Punda Maria, where current yields are projected to decrease by more than 50% in both scenarios and periods (Fig 4). Although the climate change impact increases with the worsening of scenarios, impacts under RCP6.0 are lower than those projected under RCP 4.5 for both 2030 and 2050. Unlike for sunflower, the impacts at Ofcolaco are higher than those at Syferkuil in 2050 under RCP 6.0 and RCP 8.5 but less under RCP 2.6 and RCP4.5 (Fig 4).

#### 3.3.3 Sunflower

**Fig 5:**
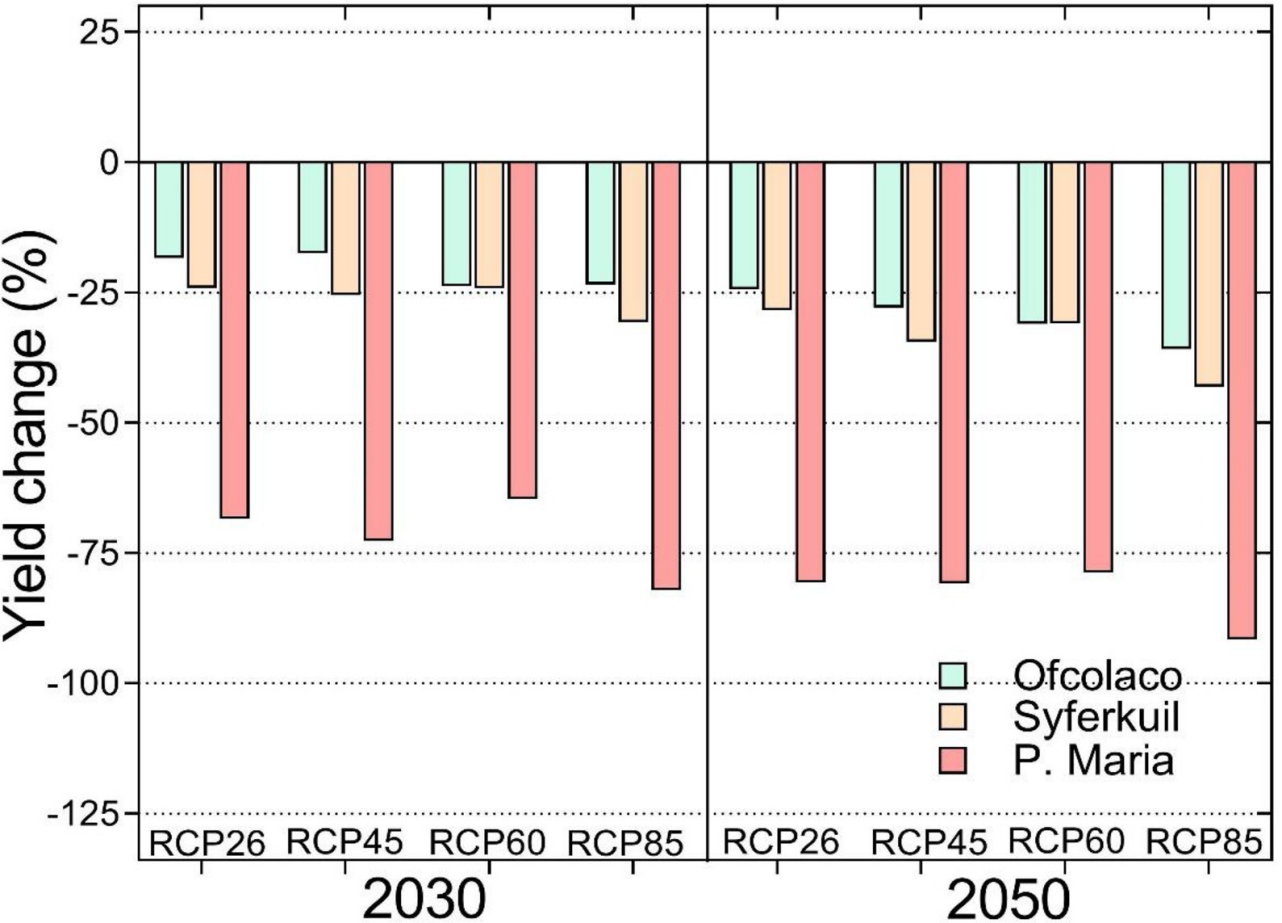
Predicted climate change impacts on sunflower yield at Ofcolaco, Syferkuil and Punda Maria, Limpopo, South Africa by 2030 and 2050 under four climate change scenarios.

Sunflower yield is projected to decrease across all sites and scenarios by 2030 and 2050. The worst climate change impacts are expected at Punda Maria, where current yields are projected to decrease by more than 80% by both 2030 and 2050 under RCP 8.5 (Fig 5). Although the climate change impacts on yield increase with the worsening of scenarios, impacts under RCP6.0 are lower than those projected under RCP 4.5. The least climate change impacts on soybean are projected for Ofcolaco whereas sunflower yield losses are at least three times less than those projected for Punda Maria. These climate change impacts on yield in Ofcolaco are less but close to those projected for Syferkuil (Fig 5). In addition, at Ofcolaco and Syferkuil the climate change impacts are higher by 2050 compared to 2030 while those at Punda Maria are similar.

#### 3.3.4 Impacts of climate change across crops and sites

**Fig 6:**
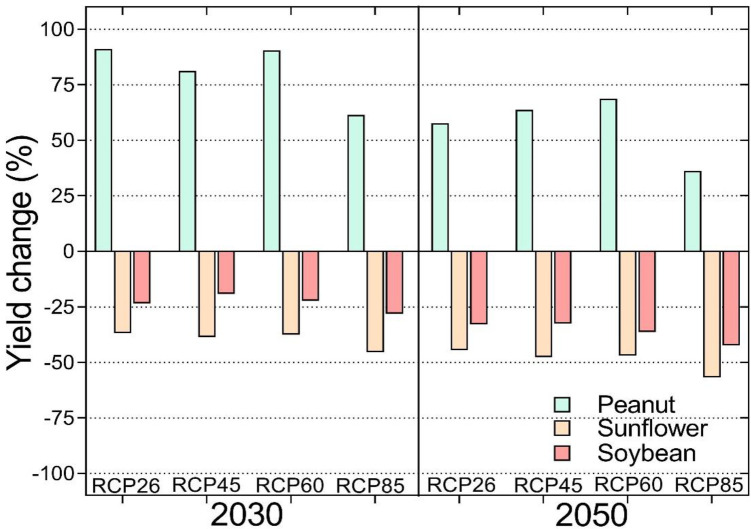
Predicted climate change impacts for peanut, sunflower and soybean across Ofcolaco, Syferkuil and Punda Maria, Limpopo, South Africa by 2030 and 2050 under four climate change scenarios.

A comparison of the climate change impacts across sites shows that groundnut yield is projected to increase under climate change while notable yield losses are projected for sunflower and soybean (Fig 6). The climate change impacts on yield are highest for RCP 8.5 for both 2030 and 2050 but the yield benefits for groundnut are least for this scenario, being highest on RCP 2.6 by 2030 and for RCP 6.0 by 2050. The results indicate that the worst impacts are expected for sunflower across all scenarios followed by soybean (Fig 6).

### 3.4 Greenhouse gas emissions

Total GHG emissions were modelled using CFT for each of the three fertilizer applications, crops and sites (Table 4). The highest GHG emissions in **(**kg CO_2_eq/ha) were observed in sunflower production across all sites. GHG emissions increased with increasing N application per hectare. However, GHG emissions in soybeans and groundnut production were notably low compared to sunflower and showed a similar pattern of increasing GHG emissions with increasing P application per hectare across all sites (Table 4).

**Table 4:** Estimated carbon footprint per unit area (kg CO_2_eq/ha) under climate change for the different crops and fertilizer regimes in Syferkuil, Ofcolaco, and Punda Maria, Limpopo, South Africa for the 2015–2050 period.

Crop	Treatment		
Levels	0 kg/ha P	30 kg/ha P	60 kg/ha
Soybeans	520.55	552.3131	584.07
Groundnut	520.55	552.31	584.07
Sunflower	531.23	1310	2200

**Fig 7:**
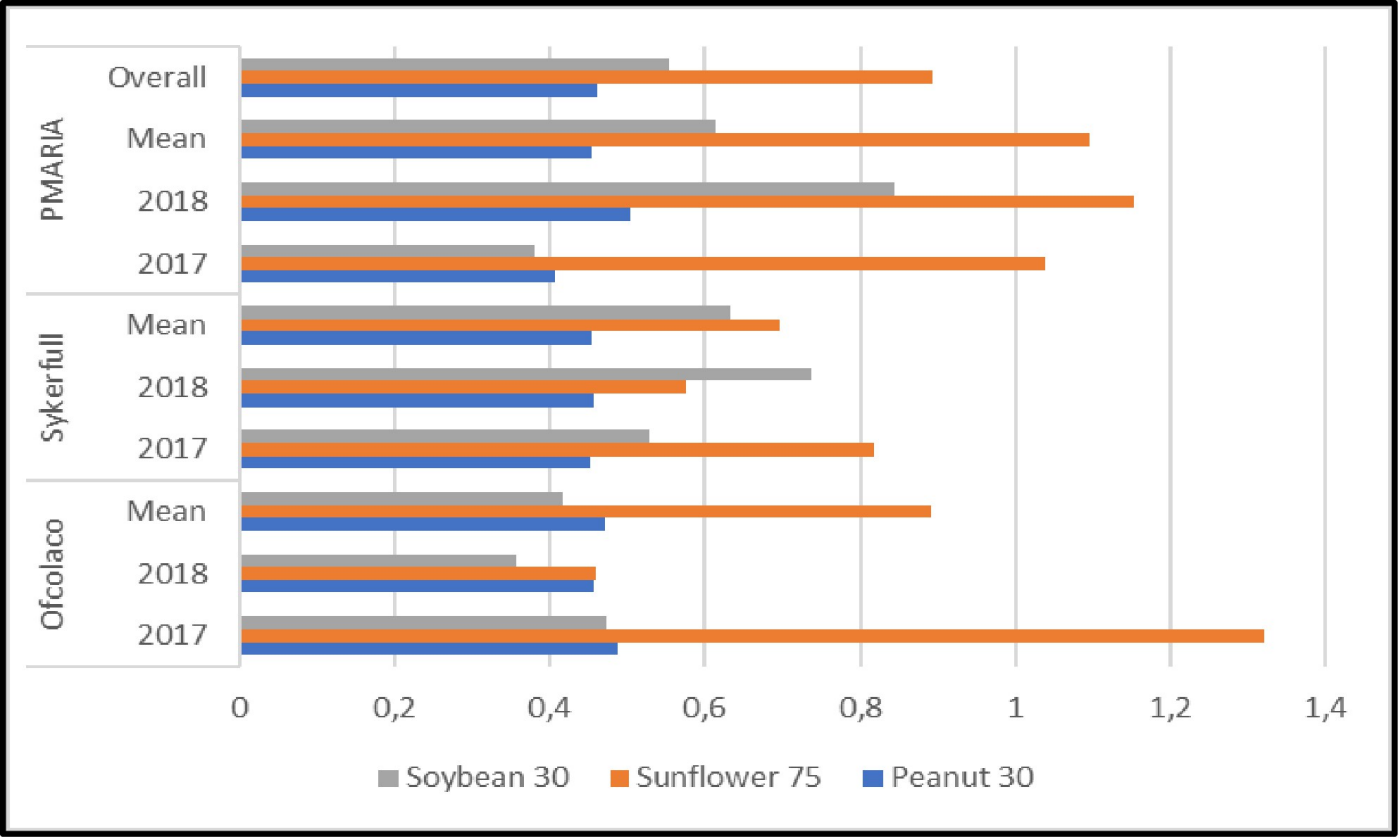
Projected changes in GHG emissions under climate change for the different crops and fertilizer regimes in Syferkuil, Ofcolaco, and Punda Maria, Limpopo, South Africa for the period 2015–2050.

The carbon footprint per unit yield (kg CO_2_eq/kg) is shown in Fig 7. Sunflower has the highest carbon footprint per unit yield across all sites with groundnut having the lowest carbon footprint per unit yield. Fig 7 shows the projected changes in GHG emissions under climate change for the different crops and fertilizer regimes in Syferkuil, Ofcolaco and Punda Maria, Limpopo, South Africa for the period 2015–2050. Positive climate change impacts in terms of reduction of GHG emissions per unit yield were observed in groundnuts at Ofcolaco and Syferkuil and for sunflower in Ofcolaco. At these two sites, all climate change scenarios show about a 50% decrease in the carbon footprint in producing groundnuts.

**Fig 8:**
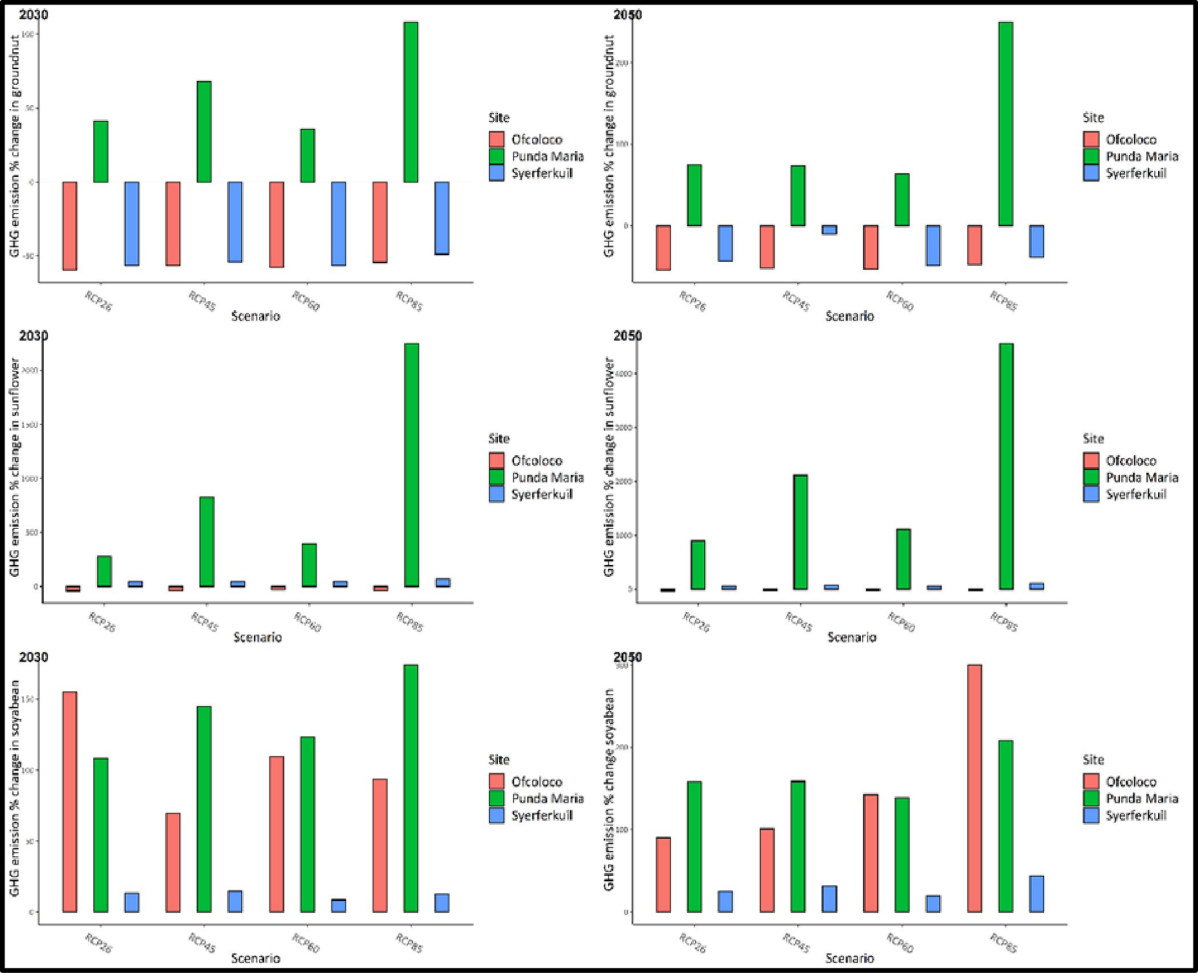
Predicted climate change impacts on GHG emission percentage change on groundnuts, sunflower and soybean production at Ofcolaco, Syferkuil and Punda Maria, Limpopo, South Africa by 2030 and 2050 under four climate change scenarios.

Surprisingly, Fig 8 shows that the carbon footprint of producing groundnuts is expected to increase by 40 to 107% in Punda Maria for the period up to 2030. Meanwhile, for the period 2050, the carbon footprint is expected to increase by 70–250%. Similarly, in Punda Maria, sunflower followed a similar trend to the one observed for groundnut with the carbon footprint expected to increase by 280–2250% up to 2030. On the other hand, for the period up to 2050 the carbon footprint is expected to increase by between 900–4450% depending on the scenario. At Ofcolaco, the positive impact of climate change is expected as the carbon footprint of sunflower is expected to decrease by 20–40% depending on the scenario for the period up to 2030 and 2050. In Syferkuil, the carbon footprint of sunflower is expected to increase by 40–70% for the period up to 2030 whereas, for the period leading to 2050, the carbon footprint is expected to increase by 55–104%. Soybean results show that climate change will result in an increase in the carbon footprint of producing soybean across all sites with significantly high increases in Punda Maria.

## 4. Discussion

The goal of this study was twofold: first, to assess the potential impact of climate change on three important oil seed crops (soybean, sunflower, and groundnut) at three sites in South Africa; Secondly, to estimate the GHG emissions from the production of these crops under climate change using modelling approaches. It is expected that the results will be important to add to the limited literature on climate change impacts on oil seed crops, to spur adaptation planning and investment, and also provide quantitative information on emissions related to the production of these crops to enhance mitigation and GHG accounting from the agriculture sector.

The DSSAT crop model requires calibration based on emergence dates, soil moisture, Leaf Area Index (LAI), biomass, phenology (sowing, germination, anthesis, and maturity dates), and grain yield and yield components [25]. One of the main challenges in crop modeling is the availability of comprehensive calibration data. This study was only calibrated based on days to flowering and grain yield for 3 crops, fertilizer treatments and 3 sites for 2016/17-2017/18 seasons, as other variables were not collected. This data limitation is common in agricultural research, and it makes it challenging to fully capture the complex dynamics of crop responses to climate change. However, the overall model calibration based on the available parameters was satisfactory as the RMSE was acceptable since it was below 30% in 70% of the crops, fertilizer treatments, and sites as seen in Moriasi et al. [26]. Furthermore, He et al. [34] in their study assess data requirements for effective model calibration and conclude that most effective model calibration is to use data from contrasting environments (at least two different seasons, at best from different sites with contrasting climates), particularly with in-season growth measurements (e.g. biomass and LAI), which could potentially reduce the simulation errors to close to the average measurement error in typical agricultural field experiments.

The outputs and recommendations of the study are therefore reliable. However, we highlight the need for the collection of many variables that can be used for model calibration. Hoogenboom et al. [25] provide a minimum data set list required for crop modelling that should be collected in experimental sites for effective model calibration and validation. Experimental field trials should therefore be designed to collect comprehensive data for crop modelling. Experimental field trials are, however, very expensive to establish and manage. Therefore, this might be challenging in sub–Saharan Africa. Hence, it might be important to establish fewer trials where sufficient modeling data is collected as opposed to many trials collecting fewer data sets. Alternatively, data for model calibration might be sourced from secondary data sources as proposed by Kephe et al. [35] as well as from institutional repositories. Unfortunately, experimental field data on oil seed crops is not widely available as there is limited research on oil seed crops in sub-Saharan Africa [36]. Due to the critical need for information on the predicted impacts of climate change on crops such as oil seed crops, there is a need for continued undertaking of crop model simulations to provide recommendations despite the limited calibration and validation. These recommendations should also be taken as general recommendations as opposed to exact recommendations.

In this study, we quantified the impacts of climate change on three important oil crops in South Africa: groundnut, sunflower, and soybean using a process-based modeling framework and climate projections. The modeling results project differential climate change impacts on oilseed crops and locations in the Limpopo province of South Africa. Some of the findings show potential for improved yield from groundnut for at least two of the three sites. Furthermore, the least impacts at the other site confirm the potential of continued groundnut production under climate change. Positive climate change response of groundnut has been reported in many other studies [37–39]. Two possible reasons explaining this positive response of groundnut to climate change. Firstly, the harvested parts of groundnut develop underground and are therefore not directly exposed to the vagaries of weather for a long time compared to other crops [40]. Secondly, groundnuts have a shorter growth period compared to other crops thereby enabling it to complete its growth cycle with minimum exposure to the environment. While production planning, area allocation and expansion targeting can be focused on crops such as groundnut which are projected to increase under climate change, other studies have indicated that climate change may affect other aspects of groundnut production such as insect pests, weeds and diseases [41], oil content [42] and other quality aspects [43, 44] which were not considered in this assessment.

Our modeling projects a decrease in soybean yield with the magnitude of the impacts varying with the scenario and site. Hao et al. [45] posit that the most likely climate change impacts on soybean yield will be through water stress or temperature-mediated increased evaporative demand at the canopy scale from warming that affects soybean water balance. Although no detailed assessment of plant water dynamics was performed in this study because of the lack of good quality soil moisture measurements, we speculate that this could be the impact pathway as the study sites relatively receive low rainfalls compared to other farming areas. Studies have shown that elevated CO_2_ levels can enhance soybean production through carbon dioxide fertilization, which is essential for photosynthesis. Increased photosynthesis is directly related to increased productivity. The negative effects of temperature and water s in many areas will, however, exceed the positive effects of CO_2_ fertilization, resulting in a negative net change [11, 46, 47]. Similar trends have been reported elsewhere; for example, Guo et al. [48] projected a 49% decrease in soybean yields by 2050 under climate change. There is a need for further studies to evaluate the crossover point where the positive impacts of increased CO_2_ are canceled by increased temperatures.

Sunflower has the highest yield potential of the three oilseed crops assessed in this study and yet is projected to be vulnerable to climate change impacts. This is significant in that sunflower is the dominant source of vegetable oil in the country and is widely grown across the country. This, therefore, means that any yield changes will have a significant influence on food and nutrition security in the country and region. The response of the sunflower crop to temperature and water deficit has been reported in the literature. For example, it is known that high temperatures increase the rate of enzymatic reactions [49] modify the structure and activity of macromolecules [50], alter the composition and structure of cell membranes, and photosynthesis as the thylakoid membrane, shape and arrangement are modified when the oxygen emitter complex in photosystem II is destroyed by heat [51, 52]. Given these effects, concerted efforts in building adaptive capacity for sunflower farmers are urgently required.

There is a need for adaptation planning to buffer projected yield losses for soybean and sunflower in South Africa. Various climate change adaptation strategies have been proffered for oilseed crops such as shifting sowing dates, selecting cultivars with high-temperature resistance, high thermal requirements and short growth periods, irrigation, and shifting production to new and more suitable areas. While these have the potential to build resilience, there is also a need to consider identifying potential barriers to their adoption to increase uptake [53]. Robust approaches are needed to sustain the productivity of existing oilseed crops and meet the challenge of food and nutrition security in the area of global climate change and these are needed urgently for already low-productivity areas such as the Limpopo province of South Africa.

In terms of GHG emissions chemical fertilizer application, land clearing and direct and indirect burning of fossil fuels contributed significantly to the carbon footprint. The results clearly showed that increased fertilizer application will result in increased GHG emissions per unit area. Climate change scenarios predict high-inter annual variation in crop yields and a trend toward a decrease in crop yields. Our modeling results show differential impacts of future climate change on the carbon footprint of oil seed crops.

Firstly, our results show that climate change may make places such as Punda Maria unsuitable for crop production for the tested crops as the environmental costs or footprint of producing oil crops may be high. Secondly, the carbon footprint of producing soybean and sunflower will generally increase across all sites. This is not surprising because the future crop yields of these two crops are expected to decline because of climate change. This should be of concern considering the need for increased production to meet the increased demand for cooking oil for the burgeoning African population. Also considering that environmental sustainability has become topical, pushing people to shift to less carbon-intensive diets, climate change mitigation will likely result in more stable crop yields and a reduction in the carbon footprint of producing these crops.

Thirdly, while future climate change has been associated with mainly negative impacts on crop production in SSA, our results show that the carbon footprint of groundnut will generally decrease mainly at Syferkuil and Ofcolaco under the four different climate change scenarios. This is not surprising because future climate change is expected to have a positive impact on groundnut yields in these areas. As a result, the carbon footprint per unit yield is expected to decrease, as such a positive impact on the future of climate change on groundnut production. Therefore, under climate change, it is better to crop groundnut in Syferkuil and Ofcolaco based on the view of reducing the environmental footprint of producing this crop. This, therefore means climate change mitigation presents complex challenges for the agricultural sector and governments in South Africa and other SSA countries who face contradicting situations on the impact of climate change on various crops. There is, therefore, an urgent need to promote low-carbon agriculture. At the same time, there is a need to increase production to meet the increasing food demand for the growing population at a very low cost. Finally, our results show hotspots of GHG emissions in cropping systems and opportunities for their reduction by improving input use efficiency.

## 5. Conclusions

The study assessed the potential impact of climate change on soybean, sunflower and groundnut production in three sites during 2030, and 2050 under different scenarios. A combination of decision-making tools was used to examine the impact of climate change not only on agricultural production but also on GHG emissions per area of production for each of the crops. The uniqueness of this study lies in its efforts to link process-based crop simulation models with decision-support tools to tackle climate change challenges. The analysis combining various models facilitated the estimation of the impacts of climate change on soybean, sunflower and groundnut production as well as the amount of GHG emission per production. The study began with model calibration which have been much improved through the use of additional variables. This highlights the need for collection in experiments of minimum agronomic data sets that can also be used for many purposes such as crop modelling. The results showed that the tools used for the study have sufficient predictive power to act as an early warning system to both stakeholders and policymakers. These can potentially help the farmers to select suitable farm management practices for agriculture development. These results could also guide policymakers to come up with better policies to assist farmers in their combat against climate change and help them reduce their environmental impact without affecting their productivity. This also increases refined available information for policymakers regarding potential trends and climate change adaptation in oil seed productivity in Southern Africa. Policies put in place should be better targeted to supporting oil seed farmers.
